# Factors and Conditions That Impact Electroporation of Clostridioides difficile Strains

**DOI:** 10.1128/mSphere.00941-19

**Published:** 2020-03-04

**Authors:** Disha Bhattacharjee, Joseph A. Sorg

**Affiliations:** a Department of Biology, Texas A&M University, College Station, Texas, USA; University of Wyoming

**Keywords:** *Clostridioides difficile*, electroporation, genetics, transformation

## Abstract

An important risk factor for acquiring Clostridioides difficile infection is antibiotic use. Therefore, a detailed knowledge of the physiology and the virulence factors can help drive the development of new diagnostic tools and nonantibiotic therapeutic agents to combat these organisms. Several genetic systems are available to study C. difficile in the laboratory environment, and all rely on stably replicating or segregationally unstable plasmids. Currently, the transfer of plasmids into C. difficile can only be performed by conjugation using Escherichia coli or Bacillus subtilis as conjugal donors. Here we report a method to introduce plasmid DNA into C. difficile using electroporation and test factors that might contribute to higher transformation efficiencies: osmolyte used to stabilize weakened cells, DNA concentration, and recovery time postelectroporation. Depending on the C. difficile strain and plasmid used, this transformation protocol achieves between 20 and 200 colonies per microgram of DNA and is mostly influenced by the recovery time postelectroporation. Based on our findings, we recommend that each strain be tested for the optimum recovery time in each lab.

**IMPORTANCE** Understanding the underlying biology of pathogens is essential to develop novel treatment options. To drive this understanding, genetic tools are essential. In recent years, the genetic toolbox available to Clostridioides difficile researchers has expanded significantly but still requires the conjugal transfer of DNA from a donor strain into C. difficile. Here we describe an electroporation-based transformation protocol that was effective at introducing existing genetic tools into different C. difficile strains.

## Abstract (precis)

Understanding the underlying biology of pathogens is essential to develop novel treatment options. To drive this understanding, genetic tools are essential. In recent years, the genetic toolbox available to Clostridioides difficile researchers has expanded significantly but still requires the conjugal transfer of DNA from a donor strain into C. difficile. Here we describe an electroporation-based transformation protocol that was effective at introducing existing genetic tools into different C. difficile strains.

## INTRODUCTION

Infections caused by *Clostridioides* (*Clostridium*) *difficile* (1) can be difficult to treat and led to nearly 500,000 infections with an associated 29,000 fatalities in 2011 (2). Additionally, treatment and management of C. difficile-infected patients result in costs of nearly $6 billion to the United States health care system annually (3). Antibiotic use is the greatest risk factor for the onset and the recurrence of C. difficile infection due to the disruption of the normal colonic microbiome, which protects against C. difficile invasion (4). The development of novel, narrow-spectrum antibiotics or non-antibiotic-based therapeutics to combat C. difficile infection requires a detailed knowledge of the molecular basis of virulence. Studying virulence and related pathways requires advanced tools of genetic manipulation. Historically, clostridial species, especially C. difficile, are notoriously difficult to genetically manipulate in comparison to model organisms (e.g., Escherichia coli or Bacillus subtilis). Several C. difficile genetic tools are available, including the following: (i) gene inactivation using a segregationally unstable pIP404-based E. coli-Clostridium perfringens shuttle vector (5, 6); (ii) TargeTron (or ClosTron), used widely for insertion of mobilizable group II introns into genes to produce stable insertion mutants (7, 8); (iii) Mariner transposition (9); (iv) allele-coupled exchange (ACE), using either *codA*-based or *pyrE*-based counterselection markers (10, 11); and (v) CRISPR-Cas9-mediated (12), CRISPR-Cpf1-mediated (13), and endogenous CRISPR-mediated (14) genome editing. Despite their utilities, each requires the conjugal transfer of the plasmids into the C. difficile cell using either E. coli (5) or B. subtilis (15) as a donor.

Introduction of plasmid DNA using conjugation requires counterselection against the donor strain, and DNA transfer occurs at lower frequencies for plasmids with segregationally unstable origins of replication (e.g., ACE systems) (16). Moreover, “suicide” plasmids (i.e., replication-deficient plasmids) are not feasible for delivery of heterologous sequences to the C. difficile chromosome due to extremely low conjugation efficiencies (10). However, recent advancements have led to increased conjugation efficiencies (17, 18). To transform such plasmids, electroporation is often used. First utilized for introducing genes into mouse lyoma cells in 1982 (19), electroporation has since been used for introducing DNA into both Gram-positive and Gram-negative bacteria (20–22), Saccharomyces cerevisiae (23), plant protoplasts (24), and a host of mammalian tissues (25). Electroporation has the additional advantage that it can be used in other recombination-based genetic systems (e.g., the lambda RED system, which requires the transformation of linear DNA fragments) (26).

In prior work, B. subtilis was shown to be transformed by plasmids during electroporation (27). B. subtilis can naturally transform linear DNA and multimeric plasmids (28) but only in certain strains (29). Moreover, electroporation has drastically improved genetics in Bacillus cereus (30) and many clostridia (31–40). In the case of C. difficile, Ackermann and colleagues reported a protocol for the clinical strain P-881 by electroporation (41). Unfortunately, for unknown reasons, this method has not been reproduced by other laboratories (5). Another attempt to electroporate C. difficile strains CD3, CD6, and CD630 using a protocol developed with Clostridium beijerinckii NCIMB 8052 also was not successful (5).

Here we report a method to introduce plasmid DNA into C. difficile using electroporation. We also tested the factors that contribute to higher transformation efficiencies (e.g., osmoprotectants, DNA concentration, and recovery time postelectroporation). The reported method was successful in transforming three different C. difficile strains: R20291 (RT027), CD630 (RT012), and JSC10 (UK1 *cspC*::*ermB*; RT027).

## RESULTS

### Initial success transforming C. difficile R20291 by electroporation.

**FIG 1:**
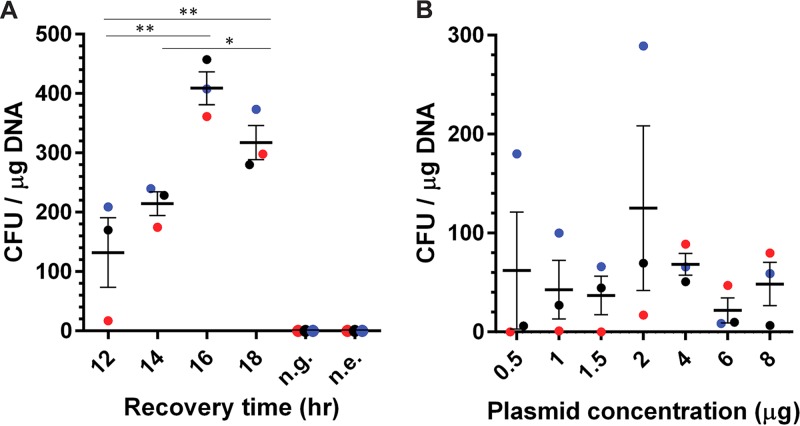
Electroporation of C. difficile R20291 competent cells grown in sorbitol-containing medium. (A) Electroporated C. difficile R20291 competent cells were recovered in BHIS medium supplemented with 500 mM sorbitol for the indicated times and plated onto BHIS medium supplemented with thiamphenicol. n.g., no glycine; n.e., no electroporation. *, *P* < 0.05; **, *P* < 0.01. (B) C. difficile R20291 competent cells transformed using different concentrations of pJS116 and recovered for 16 h. No significant differences were observed between samples. Bars represent the averages from three independent experiments (data points represent the individual experiments and are colored by competent-cell batch), and error bars represent the standard errors of the means.

Prior studies have reported successful transformation of C. difficile cells by electroporation. However, for unknown reasons, these methods were not widely adopted to introduce DNA into C. difficile cells. As an initial foray into developing an electroporation protocol that might work between different C. difficile strains, C. difficile R20291 cells were grown overnight in liquid BHIS medium (brain heart infusion [Difco] supplemented with 5 g/liter of yeast extract and 0.1% l-cysteine). Competent cells were then generated by back diluting the culture to an optical density at 600 nm (OD_600_) of 0.1 in 10 ml of liquid BHIS medium supplemented with 1% glycine (as a cell wall-weakening agent) and 500 mM sorbitol (as an osmoprotectant). At high concentrations, glycine can be incorporated into cell wall precursors in place of d-alanine, thereby reducing peptidoglycan cross linkages (42). Even though other agents, such as d/l-threonine, ampicillin, and Tween 80, have been used to weaken cell walls during growth (29, 35, 37, 43), we only tested glycine in this study. To generate competent cells, the cells were grown for approximately 12 to 16 h to an OD_600_ of 0.5. The culture was again back diluted into 40 ml of BHIS medium supplemented with sorbitol and glycine to an OD_600_ of 0.1 and grown to an OD_600_ of 0.5. Subsequently, the cell culture was washed three times with ice-cold SMG buffer (0.5 M d-sorbitol, 0.5 M mannitol [BDH], and 15% glycerol [Fisher Scientific]) (29). Approximately 2 μg of the C. difficile shuttle vector, pJS116, was added to the competent cells (44). The cells were then electroporated in a 0.2-cm electroporation cuvette at 1,250 V, 25 μF, and 200 Ω. The transformed cells were introduced into the anaerobic chamber, and recovery medium was added. The next day, the cells were pelleted and resuspended, and the entire growth was plated on BHIS medium supplemented with thiamphenicol. Surprisingly, several colonies grew. A DNA-only (no electroporation) negative control did not yield any transformants, nor did cells grow in the absence of glycine (Fig. 1A).

### Transformation efficiency is influenced by recovery time and DNA concentration.

We used the protocol described above to determine the number of hours postelectroporation required to yield the maximum number of colonies on selection plates. As shown in Fig. 1A, approximately 100 to 200 colonies grew after 12 h of growth posttransformation. This number increased up until 16 h posttransformation, when we observed approximately 400 colonies per microgram of DNA transformed. After 16 h of growth, the number of transformed cells began to decrease (Fig. 1A). Based on these data, we chose to use a 16-h recovery time for subsequent C. difficile R20291 transformations.

Next, to determine the concentration of the pJS116 vector that could yield the best transformation, we transformed different plasmid concentrations into C. difficile R20291 competent cells and recovered the transformed cells for 16 h (Fig. 1B). We observed that between 2 and 4 μg of plasmid DNA yielded the most consistent number of colonies, even though the results were not significantly different between the plasmid concentrations. Transformations with plasmid concentrations below 2 μg did not consistently yield colonies (Fig. 1B). For clarity, data points for each batch of competent cells are colored. At the 4-μg DNA concentration, the most consistent CFU were observed among the three competent-cell batches.

### Sucrose is superior to sorbitol when generating C. difficile electrocompetent cells.

In order to determine whether the above-described protocol was applicable to other C. difficile strains, we grew C. difficile CD630 and JSC10 under the conditions listed above. Unfortunately, neither of the strains produced any colonies after transformation. A possible reason for this observation could be the use of sorbitol to generate the weakened cells. Based on prior reports (41), we reasoned that sucrose may provide different results. C. difficile R20291 was grown for 8 to 10 h in BHIS medium and then inoculated to an OD_600_ of 0.1 in 10 ml of BHIS supplemented with 270 mM sucrose and 1% glycine. Cells were grown for 14 to 16 h to reach an OD_600_ between 0.5 and 0.8. Subsequently, the 10-ml culture was used to inoculate 40 ml of BHIS medium supplemented with 270 mM sucrose and 1% glycine at an OD_600_ of 0.1 and grown to an OD_600_ of 0.5. The culture was then washed with SMP buffer (270 mM sucrose, 0.938 mM MgCl2·6H_2_O [VWR], 7 mM sodium phosphate dibasic [VWR], 15% glycerol at pH 7.4) and transformed as described above.

**FIG 2:**
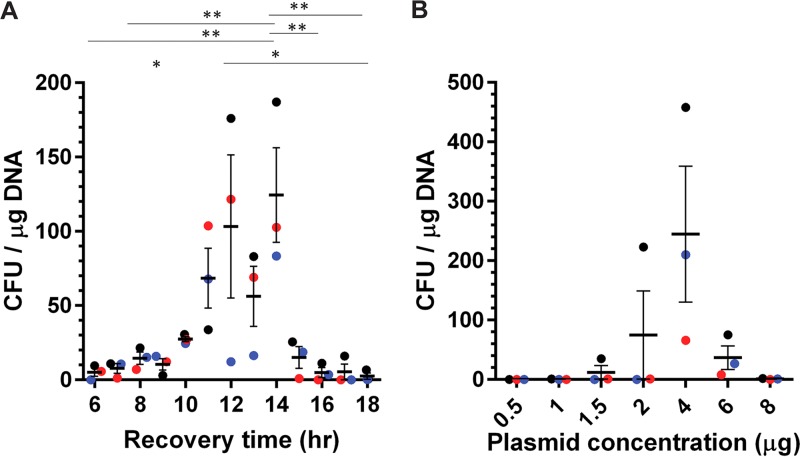
Electroporation of C. difficile R20291 competent cells grown in sucrose-containing medium. (A) Electroporated C. difficile R20291 competent cells were recovered in BHIS medium supplemented with 270 mM sucrose for the indicated times. *, *P* < 0.05; **, *P* <0.01. (B) C. difficile R20291 competent cells were electroporated with different concentrations of pJS116 and recovered in BHIS medium supplemented with 270 mM sucrose for 11 h. No significant differences were observed between samples. Bars represent the averages from three independent experiments (data points represent the individual experiments and are colored by competent-cell batch), and error bars represent the standard errors of the means.

Because this new medium may influence the time required to recover postelectroporation and/or the concentration of the plasmid required to achieve the highest number of transformants, we again tested these variables (Fig. 2). To determine the number of hours required to recover the transformed cells, we electroporated C. difficile R20291 with 4 μg of pJS116 and recovered the transformed cells for 6 to 18 h (Fig. 2A). We observed the greatest number of colonies when the transformed cells were recovered for 11 to 14 h. As was the case with the plasmid concentrations in Fig. 1B, we observed no statistical significance between different plasmid concentrations in this new medium and buffer (Fig. 2B). However, at 4 μg, we observed more consistent numbers of colonies between the three different competent cell preparations, suggesting that 4 μg may yield the most consistent results going forward.

### Growth in 1% glycine and subsequent transformation do not introduce mutations into the C. difficile genome and generate deformed C. difficile cells.

In order to understand if the electroporated cells were indeed C. difficile R20291 and not a thiamphenicol-resistant contaminant, genomic DNA from three biological replicates (2 from transformations with sorbitol as the osmolyte [Fig. 1] and 1 from a transformation with sucrose as the osmolyte [Fig. 2]) were prepared and sent for genome resequencing. As a control, the parental C. difficile R20291 strain was also sent for genome resequencing. The sequences of the transformed isolates did not deviate from that of the parental strain, indicating that the transformed cells were indeed the C. difficile R20291 strain and that the protocol to generate electrocompetent cells and subsequent transformation does not introduce mutations into the C. difficile genome.

**FIG 3:**
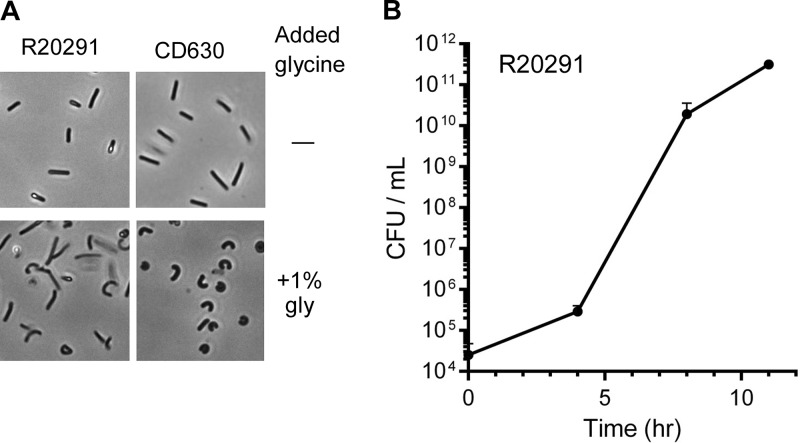
C. difficile competent-cell properties. (A) C. difficile R20291 and CD630 competent cells were prepared as described in Materials and Methods and compared to cells grown in BHIS medium alone using phase-contrast microscopy. (B) C. difficile R20291 competent cells were suspended in recovery medium and samples were taken at the indicated time points to assess viable cells on BHIS medium. Data points represent the averages from three independent experiments, and error bars represent the standard errors of the means.

To understand how growth under high glycine concentrations affects C. difficile physiology, we imaged C. difficile R20291 and CD630 cells grown in rich medium alone or in medium supplemented with 1% glycine. As shown in Fig. 3A, we did not observe protoplasts. Rather, C. difficile R20291 and CD630 cells took on a curved shape in the presence of 1% glycine.

### Recovery of competent cells over time.

Because we observed both misshapen cells and bacillus-shaped cells, we tested the outgrowth of the competent-cell preparation. C. difficile R20291 competent cells were prepared as described above and recovered in media supplemented with sucrose. Subsequently, we tested the number of CFU as a function of time on BHIS medium. As shown in Fig. 3B, we observed a small increase in colony formation from time 0 to 4 h after inoculation. Because cells are plated on BHIS medium (in the absence of sucrose), these cells are likely to be bacillus shaped and not curved with weakened cell walls. Between 4 h and 8 h, we observed a large increase in the numbers of viable cells. This suggests that between these time points, the competent cells were recovering and contributing to the CFU counts.

### Transformation of C. difficile CD630 and JSC10.

**FIG 4:**
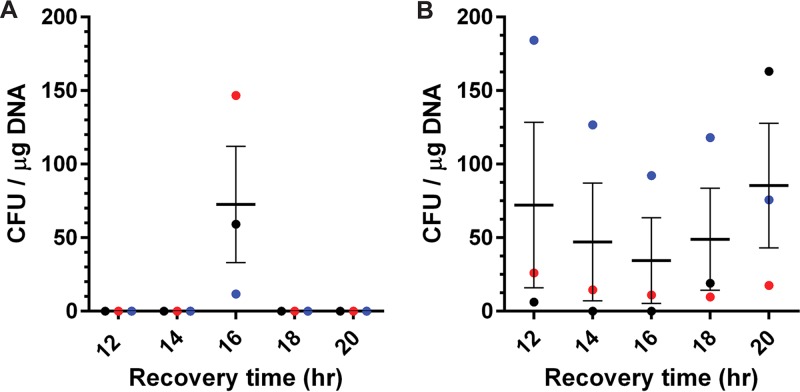
Transformation of C. difficile strains CD630 and JSC10. (A) C. difficile CD630 competent cells were electroporated with pJS116 and recovered for the indicated times. (B) C. difficile JSC10 competent cells were electroporated with pJS116. Bars represent the averages from three independent experiments (data points represent the individual experiments and are colored by competent-cell batch), and error bars represent the standard errors of the means.

Due to the success that we had in transforming competent cells stabilized with sucrose, we applied this protocol to transform C. difficile CD630 (ribotype 012) with one modification: the SMP buffer for this strain omitted 15% glycerol, as we did not recover any transformed cells when there was glycerol in the background. Due to this modification, the production of fresh competent cells for every electroporation was required. Using this protocol, C. difficile CD630 required 16 h for recovery after electroporation with pJS116 (Fig. 4A), with nearly no colonies observed at any other time point. Additionally, a different C. difficile strain (JSC10 [UK1 *cspC::ermB*]) was electroporated with pJS116 and transformed colonies were recovered successfully between 12 h and 20 h (Fig. 4B).

### Freezing competent C. difficile R20291 cells reduces viability.

**FIG 5:**
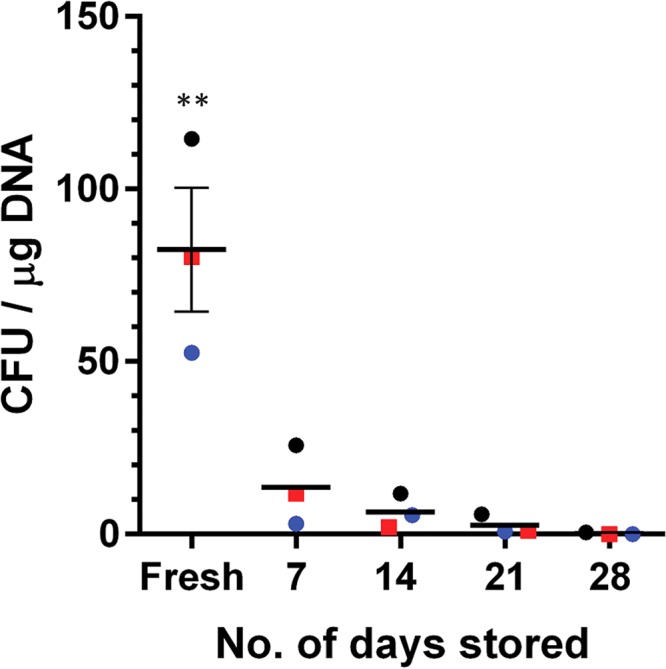
Testing the impact of –80°C storage on eletrocompetence. Competent C. difficile R20291 cells were frozen on dry ice and stored in –80°C for the indicated number of days before thawing on ice and transformation as for Fig. 2. Bars represent the averages from three independent experiments (data points represent the individual experiments and are colored by competent-cell batch), and error bars represent the standard errors of the means. **, *P* < 0.01.

It would be helpful if competent cells could be frozen for later use. To test if C. difficile R20291 competent cells could be safely stored at –80°C, we added 15% glycerol to the SMP buffer. Competent C. difficile R20291 cells were frozen on dry ice, stored at –80°C, and tested for the ability to be transformed and recovered over a 4-week period (Fig. 5). Fresh, not frozen, competent cells yielded the highest number of colonies (Fig. 5). Over a 4-week period, we observed that the frozen competent cells yielded fewer and fewer colonies when transformed with the pJS116 vector. Thus, we recommend that C. difficile competent cells be stored for only short durations.

### The anaerobic chamber airlock does not significantly affect sucrose-prepared competent cells.

**FIG 6:**
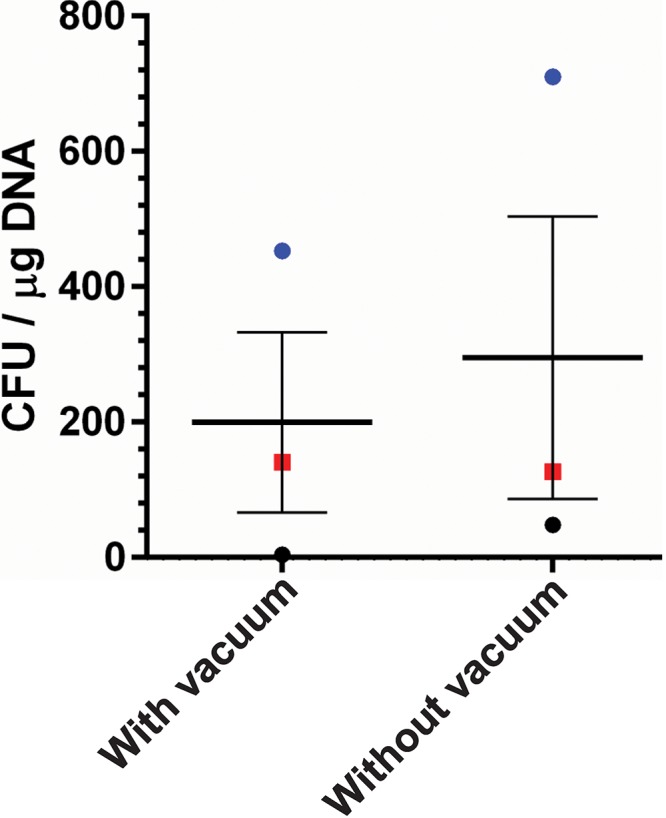
Cycling of the anaerobic chamber air lock does not influence transformation efficiency of C. difficile R20291 when grown in sucrose-containing medium. C. difficile R20291 cells were transformed as for Fig. 2. After electroporation, the cuvettes, on ice, were passed into the anaerobic chamber using the vacuum manifold or by flushing the airlock with purge gas and anaerobic gas mix for 15 s each. Bars represent the averages from three independent experiments (data points represent the individual experiments and are colored by competent-cell batch), and error bars represent the standard errors of the means.

The protocol is set up such that a part of the procedure occurs in the aerobic laboratory environment (i.e., washing and electroporation). However, in order to recover the transformed cells, postelectroporation, the cells need to be passed into the anaerobic chamber. In this process, a series of gas exchanges is done with a vacuum cycle in between each exchange. Because the cells are in a protoplast form, we hypothesized that the cells may be very sensitive to pressure changes and that the vacuum cycle, when applied to the cells, may result in the weakened cells bursting. To test this hypothesis, we passed transformed cells into the anaerobic chamber by manually flushing the airlock with a 15-s pulse of nitrogen gas and then a 15-s pulse of gas mix flushing (without a vacuum cycle). We also passed transformed cells using the normal gas exchange procedure (involving a vacuum). As shown in Fig. 6, gas exchange using the vacuum resulted in a slight decrease in transformants, but this did not achieve statistical significance.

### Transformation of other plasmids into the C. difficile R20291 strain.

**FIG 7:**
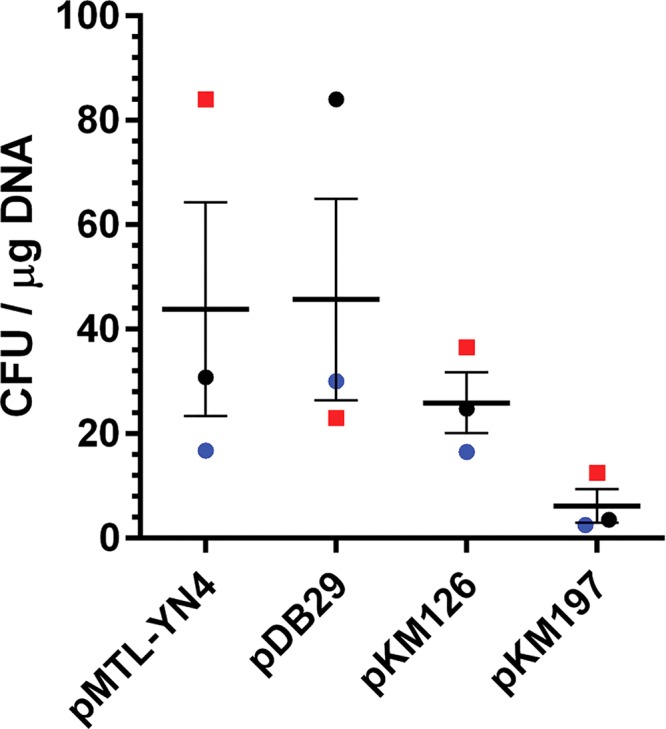
Competent C. difficile R20291 cells transformed with different plasmids. pMTL-YN4, pDB29 (a pMTL-YN4 derivative), pKM126, and pKM197 were transformed into C. difficile R20291 as described for Fig. 2. Bars represent the averages from three independent experiments (data points represent the individual experiments and are colored by competent-cell batch), and error bars represent the standard errors of the means.

**FIG 8:**
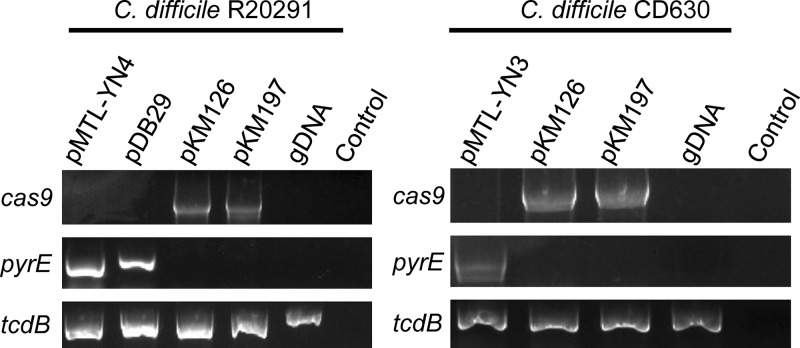
Confirming the presence of the indicated plasmids in C. difficile cells. Thiamphenicol-resistant colonies from the indicated transformation were streaked for isolation. DNA was extracted and the *C. sporogenes pyrE* allele was amplified from pMTL-YN4, pMTL-YN3, or pDB29 using oligonucleotides 5′pyrE_C_sp and 3′pyrE_C_sp. The *cas9* gene was amplified with 5′tetR_CO_cas9 and 3′COcas9 (2053). A region of C. difficile *tcdB* was amplified using 5′tcdB and 3′tcdB to confirm the presence of C. difficile. Purified genomic DNA served as a positive control for C. difficile cells, and a no-DNA-template reaction was used as a negative control.

Thus far, all the data described were obtained using an empty plasmid pJS116 vector. The described protocol would be useful if other plasmids could be introduced into C. difficile. To demonstrate that other plasmids can be transformed in C. difficile R20291 competent cells, 4-μg quantities of 4 different plasmids (pMTL-YN4, pDB29, pKM197, and pKM126) were transformed by electroporation. As shown in Fig. 7, these plasmids generated thiamphenicol-resistant colonies between 24 and 48 h after inoculation onto selective medium. To confirm that these colonies contained the respective plasmids, DNA was isolated and a portion from each plasmid was amplified by PCR (Fig. 8). The Clostridium sporogenes *pyrE* gene, carried on pMTL-YN4 and pDB29 (a pMTL-YN4 derivative), was amplified using oligonucleotides specific for this *pyrE* gene. For pKM126 and pKM197, the *cas9* gene was amplified. To confirm that the strains were C. difficile, a portion of the *tcdB* gene was amplified. Genomic DNA prepared from wild-type C. difficile R20291 did not amplify portions of the plasmids, and control reactions without template DNA did not amplify any of the tested regions.

Finally, to test if plasmids other than the empty pJS116 vector could be introduced into C. difficile CD630, we transformed pMTL-YN3, pKM126, and pKM197 into C. difficile CD630 competent cells. DNA was isolated from the resulting colonies, and as described above, regions specific to each plasmid were amplified by PCR (Fig. 8). The *C. sporogenes pyrE* gene was amplified from pMTL-YN3 but not from any of the other strains or controls. The *cas9* gene was amplified from C. difficile CD630 pKM126 and pKM197, and a portion of the *tcdB* was present in all tested strains. Control reactions without template DNA did not amplify any of the tested regions. These results indicate that the thiamphenicol-resistant colonies that arise on selective medium postelectroporation contain the indicated plasmids.

## DISCUSSION

This study provides a reliable and reproducible electroporation protocol for use with C. difficile. Previous protocols by Ackermann et al. (41) and Purdy et al. (5) have not been widely adopted in the field. The electroporation protocol described by Ackermann et al. (41) was demonstrably successful only in one clinical strain, P-881. Additionally, their protocol utilized pBAD-TOPO and pCRII-TOPO plasmids with *tcdB* insertions. These plasmids include the pBR322 origin of replication instead of a C. difficile-specific origin of replication, suggesting that their introduction may have been based on recombination with the C. difficile genome, instead of replication.

An obstacle for transformation in bacteria is presence of the cell wall, which can be circumvented by weakening the walls with glycine or penicillin. Fragility of the cell wall enhances transformation efficiency (45). Glycine is the most common additive used for electroporating B. subtilis (29), Bacillus mycoides (46), and Clostridium pasteurianum (35). We observed that C. difficile requires glycine to generate transformable cells; the absence of glycine did not produce colonies after electroporation. The addition of glycine is needed to alter the cell walls of the vegetative cells. In our experiments, 1% glycine led to the formation of curved cells but few protoplasts (Fig. 3A). Additionally, we observed that increasing the glycine concentration (i.e., to 1.5% and 2%) delayed cell culture growth by 24 to 48 h when cells were grown in 40 ml of BHIS medium supplemented with sorbitol and glycine, whereas reducing the glycine concentration to 0.75% did not produce any colonies after electroporation (data not shown).

To protect the cells from bursting, growth in medium designed to affect cell wall integrity requires an osmolyte to protect against lysis of the cell. A cell with a weakened cell wall can succumb to the turgor pressure within the cell (45). In this study, we used d-sorbitol or sucrose as an osmoprotectant. d-Sorbitol was previously used for electroporating B. subtilis (29, 47), and in prior work, our lab used d-sorbitol as an osmoprotectant to show that C. difficile and Paraclostridium bifermentans mechanosensing channels are involved in the release of dipicolinic acid from the spore core during germination (48). Though C. difficile R20291 grown in sorbitol-containing medium could be transformed by electroporation, we could not obtain any transformants from the C. difficile CD630 or JSC10 strain. However, we could obtain transformants when 270 mM sucrose was used as an osmoprotectant for all three strains tested in this study, suggesting that this osmolyte may work better than sorbitol in other C. difficile strains.

One of the important factors that influence the transformation efficiency is the recovery time in liquid medium postelectroporation (35, 45). With too short a time, the transformed cells would not have the opportunity to recover (i.e., rebuild the cell wall and divide), but recovering for a longer time could risk losing the plasmid from the transformed cells due to a lack of selective pressure on the antibiotic resistance gene. In a previous study with *C. pasteurianum*, the authors observed maximum transformation efficiency at 16 h posttransformation (35). In this study, we found that recovery time was dependent on what osmolyte was used during growth in 1% glycine. For sorbitol, the recovery time was 16 h, similar to the case with *C. pasteurianum*, but with sucrose, the recovery time was reduced to 11 to 14 h (35). Even though we observed the maximum number of colonies at 14 h of recovery, there was a sharp drop in efficiency by 15 h. To ensure that we could reliably achieve transformation, we used 11 h (where it starts to peak) for the rest of the experiments involving sucrose as an osmolyte. C. difficile CD630 maximally recovered at 16 h only; none of the other times produced colonies. Additionally, C. difficile JSC10 recovered at all the recovery times, maximally at 12 h. A variety of reasons, including growth conditions, chamber conditions, and the strain itself, could contribute to the differences observed in recovery times for the different strains.

Higher plasmid concentrations increase transformation efficiency until saturation is achieved, and this concentration is species dependent (45). We observed that the transformation efficiency with various plasmid concentrations depends on competent-cell preparation. However, on an average, higher plasmid concentrations ensured that some colonies were observed on the selection plates regardless of the osmoprotectant used to generate the competent cells or recover the transformed cells. For most experiments, we used ∼4 μg of plasmid DNA. Larger amounts of DNA led to diminishing returns on transformation, similar to what is observed in Chromobacterium violaceum (49).

It is possible that our results are due to “jackpot”-like events during different competent-cell preparations or the expansion of transformed cells (siblings) during recovery. Clearly, some competent-cell preparations yielded higher or lower numbers of transformed cells. This suggests that the growth of C. difficile in 1% glycine and subsequent washing steps are the critical components to this protocol and it is at these steps that a “good batch” of competent cells is generated. As shown in Fig. 3B, there is a small amount of growth at early time points in recovery (likely due to noncompetent cells) but a large expansion of CFU between 4 and 8 h of recovery. This suggests that there are likely siblings within our recovered cells. Because of this, the method described herein would need to be optimized before being used for transformation of a DNA library.

In this study, we mostly used pJS116 for the experiments (an empty B. subtilis*-*C. difficile shuttle vector). However, and importantly, we could electroporate the empty vector pMTL-YN4 (an ACE vector [11]), the pMTL-YN4 vector modified to have the *cspC_G457R_* mutant allele (pDB29), and two CRISPR-Cas9 mutagenesis plasmids (Fig. 7) (12) and detect these plasmids by PCR in the transformed strains (Fig. 8). These results indicate that the protocol described here can be used for the transformation of useful C. difficile plasmids. However, since many parameters may affect transformation efficiency, special attention should be paid to the parameters discussed in this protocol (i.e., hydrogen and H_2_S abundance [chamber conditions], DNA concentration, recovery time, and media used), and these may need adjustment depending on the strain used and conditions within the anaerobic chamber.

## MATERIALS AND METHODS

### Bacterial strains and growth conditions.

C. difficile R20291, CD630, and JSC10 (UK1 *cspC::ermB*) were grown in a Coy Laboratory (Grass Lake, MI) model B anaerobic chamber maintained at 37°C under anaerobic conditions (85% nitrogen, 5% carbon dioxide, >3.6% hydrogen, and an H_2_S removal system [Coy Laboratories]) on BHIS medium (brain heart infusion [Difco] supplemented with 5 g/liter of yeast extract and 0.1% l-cysteine) or, where indicated, BHIS medium supplemented with either 500 mM d-sorbitol (VWR) or 270 mM sucrose (Alfa Aesar) at pH 7.4. Antibiotic was added as needed (10 μg/ml of thiamphenicol). E. coli DH5α [F^–^ *endA1 glnV44 thi-1 recA1 relA1 gyrA96 deoR nupG purB20* φ80d*lacZ*ΔM15 Δ(*lacZYA-argF*)*U169 hsdR17*(r_K_^–^ m_K_^+^) λ^–^] was grown on LB medium at 37°C, and antibiotic was added as required (20 μg/ml of chloramphenicol).

### Plasmid construction.

A list of the plasmids used in this study can be found in Table 1. Plasmid pJS116, a C. difficile shuttle vector modified to include the Tn*916 oriT*, was used for the majority of the study as an empty-vector control (44). Plasmid pMTL-YN4 (an ACE vector) has been described previously (11). To introduce the *cspC_G457R_* allele (44) into pMTL-YN4, the C. difficile *cspC_G457R_* allele was amplified using primers 5′cspC_ger11_gp and 3′cspC_ger11_gp (Table 1) from the genome of the EMS mutant isolated by Francis et al. (44). The upstream 1-kb homology region was amplified from the C. difficile R20291 genome using primers 5′YN4_ndeI_ger1 and 3′allele_ger11_gp, and the downstream 1-kb region from the mutation was already included in the *cspC*-amplified region. Both these inserts were Gibson assembled into pMTL-YN4 cut with NdeI and XhoI and transformed into E. coli DH5α to generate pDB29. The pKM197 and pKM126 plasmids are CRISPR-Cas9 mutagenesis plasmids that introduce *pyrE* deletion mutations in C. difficile R20291. The previously published CRISPR-Cas9 *pyrE* targeting plasmid, pJK02 (12), was modified by replacing *traJ* with the Tn*916 oriT* for B. subtilis-based conjugation by amplification from pJS116 using primers 5′Tn916ori and 3′Tn916ori. The resulting fragment was introduced into pJK02 by Gibson assembly at the ApaI site and transformed into E. coli DH5α to generate pKM126. To replace the tetracycline-inducible system, the xylose-inducible promoter (50) was PCR amplified from pIA33 using primers 5′pyrE_HR_xylR 2 and 3′cas9_Pxyl 2, inserted by Gibson assembly into pKM126 at the XhoI and PacI restriction sites, and transformed into E. coli DH5α to generate pKM197. The sequences of all plasmid inserts were verified by DNA sequencing.

**TABLE 1:** Plasmids and oligonucleotides used in this study

Plasmid or oligonucleotide	Description or sequence	Reference
Plasmids		
pJS116	B. subtilis-C. difficile shuttle vector	44
pMTL-YN4	C. difficile ACE vector	11
pDB29	*cspC_G457R_* allele cloned into pMTL-YN4	This study
pKM126	*pyrE*-targeted CRISPR-Cas9 vector; *traJ oriT*	12
pKM197	*pyrE-*targeted CRISPR-Cas9 vector; Tn*916 oriT*	This study
Oligonucleotides		
5′YN4_ndeI_ger1	GAAACAGCTATGACCGCGGCCGCTGTATCCATATGCTACTTTTACAGTCTTCCCTATG	This study
5′cspC_ger11_gp	CTAATCAATTATAATTTTACATAGGTTCTTATCTATAGAGTATTTGCTATCTGTTG	This study
3′allele_ger11_gp	GATTCAACAGATAGCAAATACTCTATAGATAAGAACCTATGTAAAATTATAATTG	This study
3′cspC_ger11_gp	CAGTGCCAAGCTTGCATGTCTGCAGGCCTCGAGATGGAAAAATCTTATTGTATAATT	This study
5′Tn916ori	AAGCGGAAGAGCGCCCAATACGCAGGGCCCTAACATCTTCTATTTTTCCCA	12
3′Tn916ori	TATCTACAATTTTTTTATCCTGCAGGGGGCCCCTAAAGGGAATGTAGATAAATTATTAG	12
5′pyrE_HR_xylR 2	CATTCAAAAGAAGGAAGAACATCAATGCTTCTCGAGCTAGCATAAAAATAAGAAGCCT	This study
3′cas9_Pxyl 2	TAATCCTATACTATATTTTTTATCCATTTAATTAACTCTCCTCTTTACCCTCCTT	This study
5′tcdB	TTACATTTTGTTTGGATTGGAGGTC	This study
3′tcdB	AGCAGCTAAATTCCACCTTTCTACC	This study
5′tetR_CO_cas9	CTGAGCTCAATAATACTAGGAGGTTTTTTTAATTAAATGGATAAAAAATATAGTATAGGATTAGATATAGGAAC	This study
3′COcas9 (2053)	ATAAAATTTCTATTTGCAAATCCATCAC	This study
5′pyrE_C_sp	GAGTAATATAAATGTTATAGATATATTAAAAG	This study
3′pyrE_C_sp	CTACTACCTGGTTTTACAAAAGG	This study

### Plasmid purification.

For use in transformation, the plasmids were purified by either miniprep (Thermo Scientific GeneJET plasmid miniprep kit) or Miraprep (51). Frequently, one miniprep sample did not yield a large enough amount of plasmid DNA. To circumvent this issue, approximately 5 miniprepped-plasmid solutions were combined and ethanol precipitated. One hundred microliters of autoclaved water was added to resuspend the pellet and stored at 4°C until use. Miraprep plasmid yields were high, and thus, Miraprep was used in the majority of the experiments, with the following modification. We observed a significant amount of contaminating RNA when the Miraprep-purified plasmid was run on an agarose gel. Therefore, we increased RNase A concentrations in the published protocol to 5 to 10 times the concentration listed in the work of Pronobis et al. (51).

### Electroporation.

C. difficile strains were streaked from frozen stock in an anaerobic environment kept between 3.6% and 4.2% H_2_ on BHIS agar medium supplemented with 0.1% taurocholate [BHIS(TA)] or, for the C. difficile JSC10 strain, BHIS agar medium with 30 μl of 50-mg/ml lysozyme spread on the surface. Subsequently, a single colony was struck onto BHIS agar medium 17 to 24 h before use. A single colony was then inoculated into 5 ml of BHIS liquid medium. After 8 to 12 h of growth in BHIS liquid, the culture was diluted to a final OD_600_ of 0.1 (∼300 μl) in BHIS medium supplemented with 1% glycine and 270 mM sucrose (or 500 mM sorbitol). The resulting suspension was grown to an OD_600_ of 0.5 to 0.8 (∼12 to 16 h of growth). Subsequently, this culture was diluted to a final OD_600_ of 0.1 to 0.2 (∼5 ml) into 40 ml of BHIS medium supplemented with 270 mM sucrose (or 500 mM d-sorbitol) and 1% glycine. Glycine was included as a cell wall-weakening agent and should have resulted in the generation of protoplasts (42). Cells were then grown to an OD_600_ of 0.5 to 0.8. C. difficile R20291 required ∼2.5 to 3.5 h to reach this density, and C. difficile CD630 and JSC10 each required 4 to 6 h of growth. Once the OD_600_ was reached, the ∼45-ml culture was removed from the anaerobic chamber and kept on ice for 10 min before the cells were pelleted for 12 min at 4,000 × *g* and 4°C. The supernatant was discarded. For the next steps, the cells were kept on ice or at 4°C until placed back into the anaerobic chamber for recovery, postelectroporation. For C. difficile CD630, white debris may be found near the bottom of the pellet, and we found that discarding the debris reduced transformation efficiency. Two electroporation buffers were tested: SMG (0.5 M d-sorbitol, 0.5 M mannitol [BDH], and 15% glycerol [Fisher Scientific]) and SMP (270 mM sucrose, 0.938 mM MgCl2·6H_2_O [VWR], 7 mM sodium phosphate dibasic [VWR], 15% glycerol at pH 7.4). For transformation of C. difficile CD630 and JSC10 cells, the glycerol was omitted from SMP. The pelleted competent cells were washed three times in 10 ml of electroporation buffer (outside the anaerobic chamber), with gentle mixing of the pellet using the 10-ml pipette. The cells were kept on ice at all times. After decanting the last wash, the pellet was gently mixed into 2 ml of electroporation buffer using a blunt 1-ml pipette tip (the end of the pipette tip was removed with scissors). The ice-cold plasmid solution (∼4,000 ng in a total volume of 5 to 10 μl) was mixed into the competent cells in a 1.5-ml Eppendorf tube that had been kept at –20°C prior to mixing. The plasmid-cell mixture was then added to ice-cold 0.2-cm electroporation cuvettes (Gene Pulser/MicroPulser electroporation cuvettes; Bio-Rad). The plasmid-cell mixture was electroporated at 1,250 V, 25 μF, and 200 Ω in a Bio-Rad Gene Pulser Xcell. Immediately after electroporation, the electroporation cuvettes, still on ice, were introduced into the anaerobic chamber without using a vacuum manifold for gas exchange (the airlock was manually flushed [15-s purge gas and 15-s gas mix]). In the anaerobic chamber, the cells were flooded with 1 ml of prereduced BHIS medium supplemented with either d-sorbitol or sucrose (pH 7.4) and then added to 4 ml of the same medium (5-ml total volume). After growing for the time illustrated in Fig. 1 and 3, the 5-ml culture was centrifuged for 12 min at 4,000 × *g* and 4°C and the entire pellet was resuspended and plated on BHIS agar medium supplemented with thiamphenicol. The colonies containing the plasmid appeared between 18 and 48 h after plating, depending on the strain. Following growth on plates, colonies were enumerated. To determine the amount of CFU per microgram of DNA, the total number of colonies was divided by the amount of DNA used to transform C. difficile competent cells.

### Statistics.

Statistical analysis between the column values in Fig. 1 and 5 was accomplished with a one-way analysis of variance (ANOVA) with Tukey’s test for multiple comparisons. A 99% confidence interval was set for significance (*P* < 0.01).
